# Embedding-driven physics informed neural network for predicting optical constants across materials

**DOI:** 10.1039/d6ra00706f

**Published:** 2026-05-14

**Authors:** Sakshi Choudhary, Ravi Kumar, Annapureddy Venkateswarlu, Salla Gangi Reddy

**Affiliations:** a Department of Physics, SRM University-AP Amaravati 522 240 Andhra Pradesh India sakshi.a@srmap.edu.in gangireddy.s@srmap.edu.in; b Department of Physics, National Institute of Technology Tiruchirappalli Tamil Nadu India

## Abstract

We introduce a deep learning framework for predicting the optical constants of materials, specifically the refractive index *n*(*λ*) and the extinction coefficient *k*(*λ*), as a function of wavelength. Our model utilises learnable embedding layers to encode material-specific information into a low-dimensional latent space. This embedding-driven architecture enables the network to model wavelength-dependent optical behavior using only discrete material identifiers and normalized wavelengths as input. We also develop a physics-informed extension that incorporates a differentiable reflectance loss based on the Fresnel equation at normal incidence, allowing optional enforcement of physical constraints during training. Through systematic ablation studies, we find that this reflectance term has minimal impact on predictive accuracy, suggesting that the learned embeddings alone sufficiently capture essential dispersion characteristics. The proposed model demonstrates strong predictive performance across most material classes, although comparatively lower accuracy is observed for structurally diverse oxide materials. Overall, the framework offers scalability and potential integration into optical simulations, high-throughput materials screening, and inverse design workflows.

## Abstract 2

We develop a deep learning-based prediction for refractive index and extinction coefficients using material embeddings and optional physics-informed neural networks.

## Graphical abstract

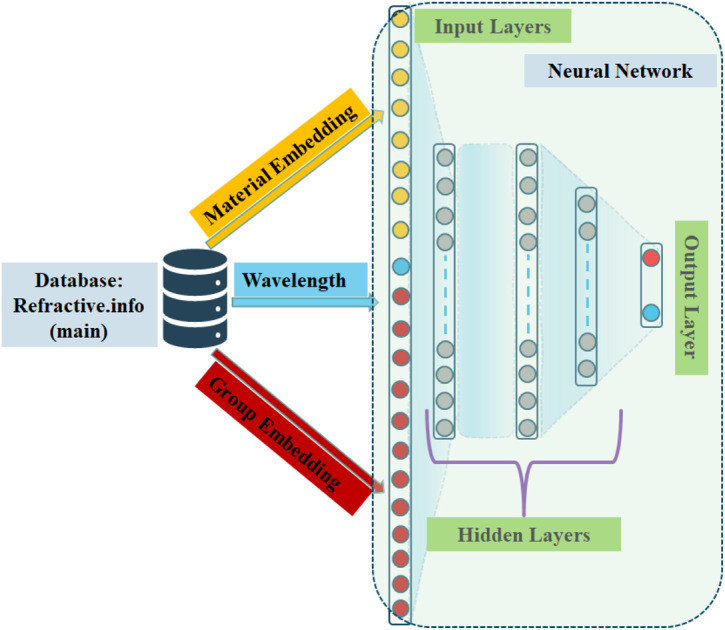

## 1. Introduction

The accurate prediction of a material's optical constants, the refractive index (*n*(*λ*)) and extinction coefficient (*k*(*λ*)), across a broad spectral range is fundamental to the design and analysis of photonic, optoelectronic, and energy-conversion systems.^1,2^ These constants govern light–matter interactions, influencing key phenomena such as reflection, transmission, absorption, and dispersion. Intrinsically, they play a pivotal role in the modelling and optimization of devices including solar cells, light-emitting diodes (LEDs), optical sensors, and metamaterials.^3,4^ The refractive index (*n*(*λ*)) characterizes how light propagates through a medium, defined as the ratio of the speed of light in a vacuum to its speed in the material. It directly affects wavefront bending, phase velocity, and optical path length. The extinction coefficient (*k*(*λ*)), on the other hand, quantifies the attenuation of light due to absorption and internal scattering within the material, contributing to intensity loss as light propagates. Together, *n*(*λ*) and *k*(*λ*), form the complex refractive index n*~*(*λ*) = *n*(*λ*) + *ik*(*λ*) which encapsulates both the dispersive and absorptive properties of the medium.^5^ Accurate knowledge of these parameters is essential for simulating interference effects, energy dissipation, and spectral selectivity in thin films, multilayers, and nanostructured optical materials.^6^

Traditional methods for determining the optical constants of a material, such as spectroscopic ellipsometry and optical reflectometry, are widely regarded as accurate and reliable.^7,8^ These techniques have become standard tools in the fields of optics and materials science, providing precise measurements of the refractive index and extinction coefficient across a range of wavelengths. However, their practical implementation is often hindered by several limitations. The measurement process typically involves intricate alignment procedures, multiple spectral acquisitions, and extensive post-processing to derive the desired parameters. Furthermore, these methods frequently rely on advanced and costly instrumentation, which may not be readily available in all research environments. Their applicability is also constrained in cases involving non-uniform or nanostructured materials, thin films, and samples with irregular surface morphology, where optical responses deviate from idealized assumptions^8^

In the context of high-throughput materials discovery and computational screening, where large libraries of candidate compositions must be rapidly evaluated, such traditional characterization methods become impractical.^9^ The combination of slow data acquisition, instrument dependency, and manual intervention poses a significant bottleneck to scaling up material analysis. These challenges have catalysed the development of computational approaches that aim to predict optical constants more efficiently. Among these, data-driven methodologies have gained prominence for their ability to model complex relationships between material properties and optical behaviour without the need for explicit physical modelling.^10,11^ When trained on high-quality experimental datasets and informed by underlying physical principles, these models offer a scalable and cost-effective alternative to experimental techniques, enabling accelerated exploration and design of novel optical and optoelectronic materials. Recent developments in machine learning and deep learning have led to the emergence of powerful frameworks capable of predicting the optical properties of materials directly from fundamental inputs such as wavelength and material identity.^10,12–16^ These approaches capitalize on the availability of large experimental and computational datasets, learning complex, nonlinear relationships that are often difficult to capture using traditional modelling techniques. When informed by physical principles and guided by domain-specific constraints, data-driven models can serve as effective surrogates for experimental measurements, offering scalable and efficient alternatives for mapping optical responses across diverse materials and spectral regions.^12,13^

A variety of machine learning algorithms, including random forests, Gaussian process regressors, and artificial neural networks, have been utilized to model the relationship between material descriptors and their optical behaviour.^16,17^ These models are typically trained on curated datasets composed of empirical or simulated refractive index and extinction coefficient values. Despite their success, conventional machine learning methods often depend heavily on feature engineering. High predictive performance generally requires the use of carefully crafted descriptors derived from elemental composition, crystallographic information, or empirical correlations.^10,18^ This dependence on manual descriptor selection can limit the model's generalization capability, particularly when applied to chemically novel or structurally ambiguous materials that fall outside the domain of the training set.

To address these limitations, recent studies have introduced embedding-based deep neural network architectures, which learn continuous latent representations of discrete input features such as material identity. Inspired by advances in natural language processing, such as Word2Vec,^19^ these embedding layers enable the model to encode semantic or functional similarity between different materials into the latent space. This approach allows for effective knowledge transfer across materials and improved generalization across complex spectral distributions. Embedding-based models have shown promising results in various domains, including molecular property prediction,^20^ materials informatics,^21^ and crystal structure-based neural networks.^12^

In this work, we propose a physics informed deep learning framework that predicts optical constants solely from three inputs: the normalized wavelength, the material identity, and group identity represented through a trainable embedding vector. Unlike conventional models, our approach requires no explicit compositional or structural descriptors. The model learns to infer optical behaviour directly from data by optimizing the latent material representation during training. The output consists of wavelength-dependent predictions of both the refractive index and extinction coefficient, spanning the ultraviolet to near-infrared (UV–NIR) spectral range. To ensure the physical plausibility of the predicted spectra, we further introduce a physics-informed variant of the model. This version incorporates a differentiable loss term derived from the Fresnel equations, which penalizes deviations between the predicted and physically consistent reflectance.^5^ The strength of this regularization is controlled by a tunable parameter *β*_refl_, allowing flexibility in balancing data-driven learning and physical constraints. Interestingly, our ablation studies suggest that the learned material embeddings are inherently expressive and capable of capturing dispersion trends, with only marginal improvements observed when adding the reflectance-based constraint. This observation indicates that the embedding space successfully internalizes relevant physical behavior from the training data itself. To further validate the expressiveness of the learned embeddings, we applied dimensionality reduction using t-distributed Stochastic Neighbour Embedding (t-SNE) to project the high-dimensional latent space into two dimensions.^22^ The resulting visualization revealed clear clustering patterns among materials belonging to similar groups. When coloured by material identity and independently by broader group labels (such as metals, dielectrics, and semiconductors), the embeddings formed distinct, physically meaningful clusters. This grouping behaviour provides strong empirical evidence that the embedding space captures not only the individual identity of materials but also shared optical characteristics at a functional level.

In summary, our method provides a fully differentiable, end-to-end learning framework for the high-fidelity interpolation of optical constants. By successfully mapping the relationship between wavelength and material identity through latent embeddings, his approach offers a computationally efficient tool for spectral data refinement and data-driven inverse design of photonic materials.

## 2. Methods

### 2.1 Dataset and overview

The dataset employed in this study was sourced from the RefractiveIndex.info database,^23^ which compiles experimentally measured optical constants, namely, the refractive index *n*(*λ*)and extinction coefficient *k*(*λ*) from a wide array of peer-reviewed studies and handbooks. These constants are typically derived *via* spectroscopic ellipsometry or optical reflectometry and are reported as functions of wavelength across the ultraviolet (UV), visible (VIS), and near-infrared (NIR) spectra. To ensure high-quality and representative learning data, only materials with smooth, continuous spectral profiles spanning the 0.2–1000 µm range were retained. Also, instances with *n* ≤ 0.1 or *k* < 0 and samples with missing values in *n*(*λ*), *k*(*λ*), or *λ* were discarded, as these are considered non-physical or missing data. Data cleaning was performed to remove non-physical entries, such as negative or missing values, while maintaining the physical diversity of the dataset. No upper-bound filtering was applied to the refractive index *n*(*λ*) or extinction coefficient *k*(*λ*). Consequently, the dataset retains high-magnitude values which are physically representative of specific regimes, such as the strong free-carrier (Drude-like) response in metals and phonon resonances in polar crystals within the infrared and far-infrared regions. Each material was assigned a unique material identifier (material_id) for substance-level distinction, along with a categorical group label (group_id) denoting its broader optical classification: metal, semiconductor, dielectric *etc.* Rather than using fixed one-hot encodings, both identifiers were mapped into learnable embedding spaces, enabling the network to capture latent similarities among materials and class-specific optical behaviour. For instance, semiconductors typically display well-defined absorption edges, while metals exhibit Drude-like dispersion; such relationships are more effectively represented through embeddings than rigid one-hot vectors. After cleaning, the final dataset included approximately 485 314 samples from over 147 distinct materials, grouped into 6 group_id classes.

**Fig. 1:**
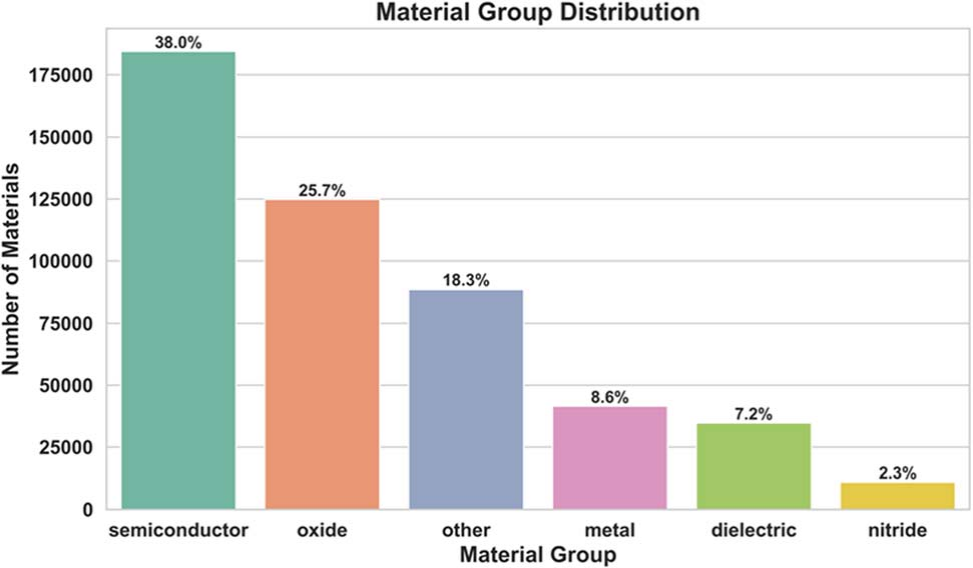
Material group distribution in the dataset. Semiconductors dominate the collection (38.0%), followed by oxides (25.7%) and other materials (18.3%). Metals (8.6%), dielectrics (7.2%), and nitrides (2.3%) constitute smaller fractions, indicating a dataset skew toward semiconductor- and oxide-based systems.

Dataset shows a clear dominance of semiconductors (38.0%) and oxides (25.7%), which together comprise nearly two-thirds of all materials as shown in Fig. 1. Other compounds (18.3%) make up a moderate portion, while metals (8.6%), dielectrics (7.2%), and nitrides (2.3%) are significantly underrepresented. This imbalance reflects the historical emphasis on semiconductors and oxides in electronic, optical, and energy applications. Meanwhile, fewer data exist for metals, dielectrics, and nitrides despite their technological significance. These disparities highlight potential areas for future dataset expansion and the need for more balanced coverage of functional material classes.

### 2.2 Feature preparation

Each sample in the dataset was represented by three primary input features: the wavelength (*λ*), which was normalized using standard scaler; the material_id, encoded as an integer index (0, 1, 2, …, 146) and passed through a 12-dimensional trainable embedding layer; and the group_id, denoting the broader class (*e.g.*, 0: metal, 1: semiconductor, 2: dielectric), which was mapped into an 8-dimensional embedding layer. These inputs were concatenated and fed into the network to jointly predict the two optical outputs: *n*(*λ*), the real part of the refractive index, and *k*(*λ*), the extinction coefficient. Importantly, no handcrafted descriptors, elemental properties, or structural features were introduced, ensuring that the framework remained a minimal-input, data-driven approach relying solely on spectral information and learned embeddings.

The specific embedding dimensions (12 for material and 8 for group) were finalized following a systematic ablation study. This study evaluated the trade-off between predictive accuracy (minimizing RMSE), generalization stability (narrowing the training and testing gap), and parameter efficiency (avoiding the curse of dimensionality). Detailed ablation results and dimensionality comparisons are provided in the SI (Section S1).

### 2.3 Data splitting strategy

The dataset was divided into three non-overlapping subsets to ensure robust training and evaluation. Specifically, 80% of the data was allocated to the training set, within which 10% was further reserved as a validation set for hyperparameter tuning and early stopping, while the remaining 20% was held out as a test set for the final performance assessment. To maintain balanced representation, stratified sampling was employed such that both material identities and group classes were proportionally preserved across all splits. Consequently, all materials are present in both training and test sets, and the evaluation reflects interpolation across wavelength for known materials. Additionally, a fixed random seed was applied to guarantee reproducibility of the modelling.

## 3. Model architecture

### 3.1 Input design

**Fig. 2:**
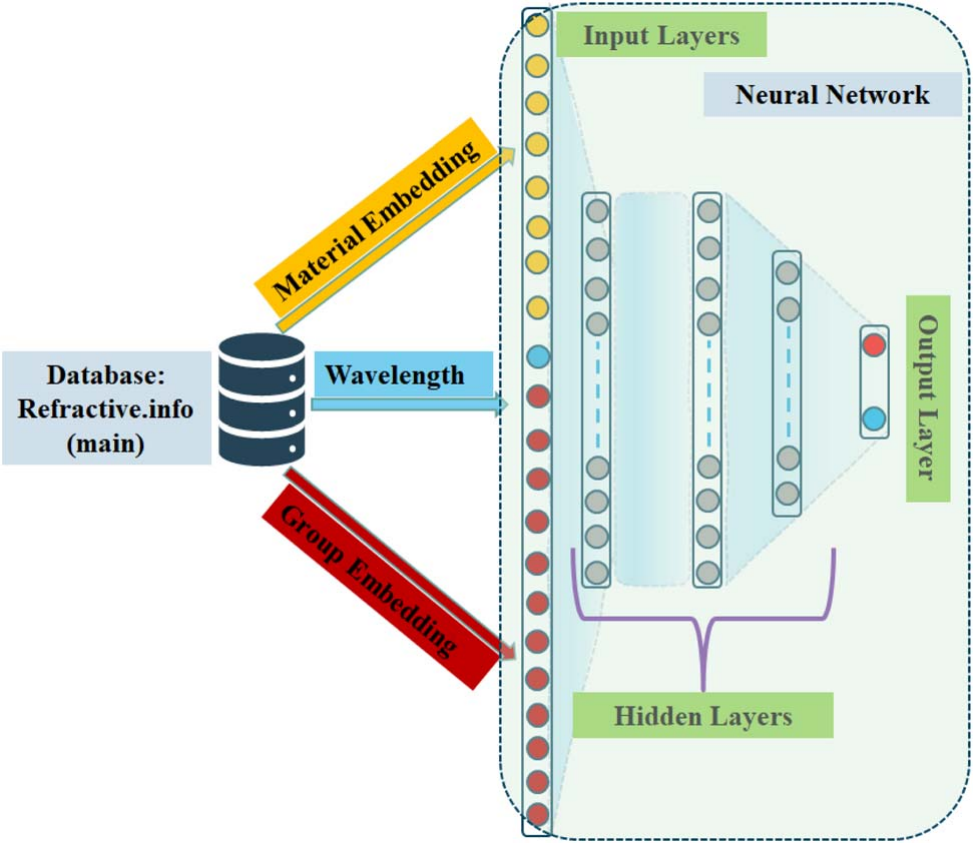
Schematic of the proposed three-input neural network for predicting optical constants. The model integrates wavelength (scalar input), material_id (12-dimensional embedding), and group_id (8-dimensional embedding) into a unified 21-dimensional representation. This vector is processed through three fully connected layers, after which the network branches into two regression heads that independently predict the refractive index *n̂* and extinction coefficient *k̂*.

The proposed architecture of neural network was shown in Fig. 2, constructed as a three-input architecture comprising: (i) the wavelength (*λ*), represented as a normalized scalar; (ii) the material identifier, mapped into a 12-dimensional embedding vector; and (iii) the group identifier, mapped into an 8-dimensional embedding vector. These three components were concatenated to form a 21-dimensional combined feature vector, which served as the input to the model.

### 3.2 Embedding layers

Let *M* and *G* denote the number of unique materials and group classes, respectively. Each discrete input index was projected into a dense latent space *via* trainable embedding layers. Specifically, the material embedding was defined by eqn (1) as:

1 *e*_*i*_ = Embedding_material_(*i*), *e*_*i*_ ∈ *R*^12^

and the group embedding was defined by eqn (2) as:

2 *g*_*j*_ = Embedding_material_(*j*), *g*_*j*_ ∈ *R*^8^

The concatenated representation was then expressed by eqn (3):

3 *x*_input_ = [*λ*, *e*_*i*_, *g*_*j*_] ∈ *R*^21^

These embeddings were learned jointly with the model parameters, enabling the network to capture both material-specific spectral responses and class-level optical trends.

### 3.3 Feature extractor and output heads

The concatenated input vector was passed through a three-layer fully connected feature extractor, consisting of: layer 1 with 64 neurons (ReLU activation), layer 2 with 64 neurons (ReLU activation), and layer 3 with 32 neurons (ReLU activation). Neither dropout nor batch normalization was applied, as the model exhibited strong generalization owing to the large training set. The shared latent representation was expressed as eqn (4), branched into two independent regression heads as:

4 *n̂* = Dense(1)(*x*), *k̂* = Dense(1)(*x*)

where *n̂* and *k̂* correspond to the predicted real part (refractive index) and imaginary part (extinction coefficient), respectively.

### 3.4 Loss function design

#### 3.4.1 Supervised loss

The primary objective was to minimize the prediction error between the model outputs and the ground-truth optical constants. A mean squared error (MSE) loss was defined by eqn (5) over both targets, refractive index *n*(*λ*), and extinction coefficient *k*(*λ*):5
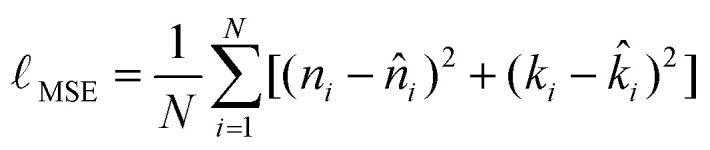
where *N* denotes the number of training samples. This formulation enforces equal importance on accurately modelling both dispersion and absorption.

#### 3.4.2 Physics-informed reflectance loss

To encourage physically consistent spectra beyond purely data-driven fitting, a physics-informed regularization term was introduced based on Fresnel reflectance at normal incidence as defined by the eqn (6):6
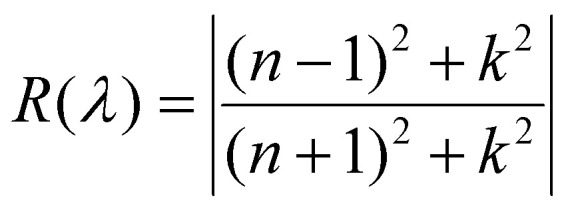


Let *R̂* be the reflectance computed from the predicted values *n̂* and *k̂*, and let *R* denote the reflectance computed from the ground-truth *n*(*λ*)and *k*(*λ*)using eqn (6). The total loss is then given by:

7 *ℓ*_total_ = *ℓ*_MSE_ + *β*_refl_ × MSE(*R*,*R̂*)

where regularization weight *β*_refl_ ∈ [0,100] balance the supervised loss with the physics-based reflectance constraint. The value of *β*_refl_ was empirically tuned, with higher values increasing the influence of reflectance consistency. A scheduler gradually increases *β*_refl_ over the first 50 epochs to avoid early suppression of learning. It is important to note that this constraint operates locally at each wavelength and does not enforce global Kramers–Kronig consistency across the spectrum; rather, it serves as a physics-informed regularization that encourages physically meaningful predictions.

### 3.5 Training protocol

The models were implemented in TensorFlow 2.15 using the Keras functional API and trained with the Adam optimizer (learning rate 0.001). Each run used up to 100 epochs with early stopping (patience 10) and Model Checkpoint to save the best weights. For the physics-informed variant, a reflectance-based loss was added, with *β*_refl_ gradually increased from 0 to its target value over the first 50 epochs using a custom scheduler. All experiments were performed on a standard laptop. To ensure reproducibility, random seeds were explicitly set across NumPy, TensorFlow, and Python environments.

### 3.6 Model evaluation and visualization

#### 3.6.1 Quantitative evaluation

Evaluating the predictive performance of the model requires metrics that not only measure numerical accuracy but also reflect the physical significance of optical constants. Since the refractive index is expressed as two distinct quantities, real component (*n*(*λ*)), describing dispersion and imaginary component also known as extinction coefficient (*k*(*λ*)) capturing absorption, each was assessed separately using three complementary statistical measures.

##### 3.6.1.1 Mean absolute error (MAE)

Mean Absolute Error (MAE) quantifies the average magnitude of prediction errors without considering their direction defined by eqn (8). In the context of optical materials, this provides an easily interpretable measure of how far the predicted refractive index values deviate from experimentally reported ones on average. A low MAE indicates that the model consistently tracks real-world optical trends with minimal deviation.8
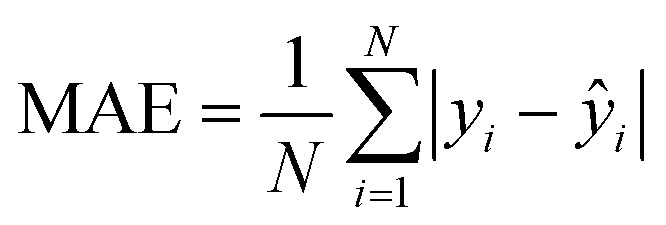


##### 3.6.1.2 Mean squared error (MSE)

Mean Squared Error (MSE) penalizes larger deviations more heavily than MAE, making it a sensitive measure for detecting rare but significant prediction failures. This is particularly important in optical physics, where even small inaccuracies at certain wavelengths can lead to disproportionately large errors in downstream calculations of reflectance, transmittance, or absorptance. By minimizing MSE, the model learns to avoid sharp inconsistencies that could compromise physical plausibility which is governed by the below eqn (9):9
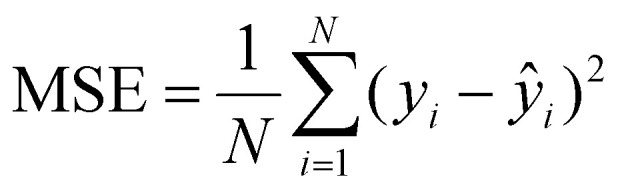


##### 3.6.1.3 Coefficient of determination (*R*^2^)

The *R*^2^ score evaluates how well the model captures the variability in the data as defined in the eqn (10). In this application, a high *R*^2^ indicates that the learned mapping from material embeddings and wavelength to optical constants reproduces not only pointwise accuracy but also the global dispersion and absorption behaviour of the material across the spectrum.10
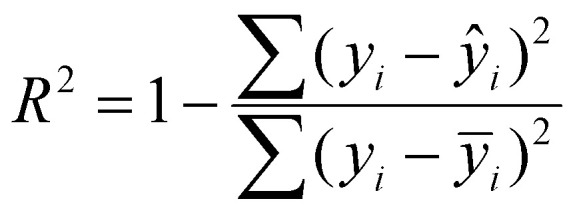
where 
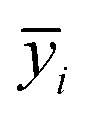
 is the mean of the observed target values. Metrics were computed separately for *n* and *k*, using the held-out test set. By combining these metrics, the evaluation framework balances local accuracy (captured by MAE), robustness to large deviations (captured by MSE), and global trend fidelity (captured by *R*^2^). This comprehensive assessment ensures that the model is not only statistically reliable but also physically meaningful, which is essential when deploying predictions for photonic design, material discovery, or optical simulations.

#### 3.6.2 Embedding analysis *via* t-SNE

**Fig. 3:**
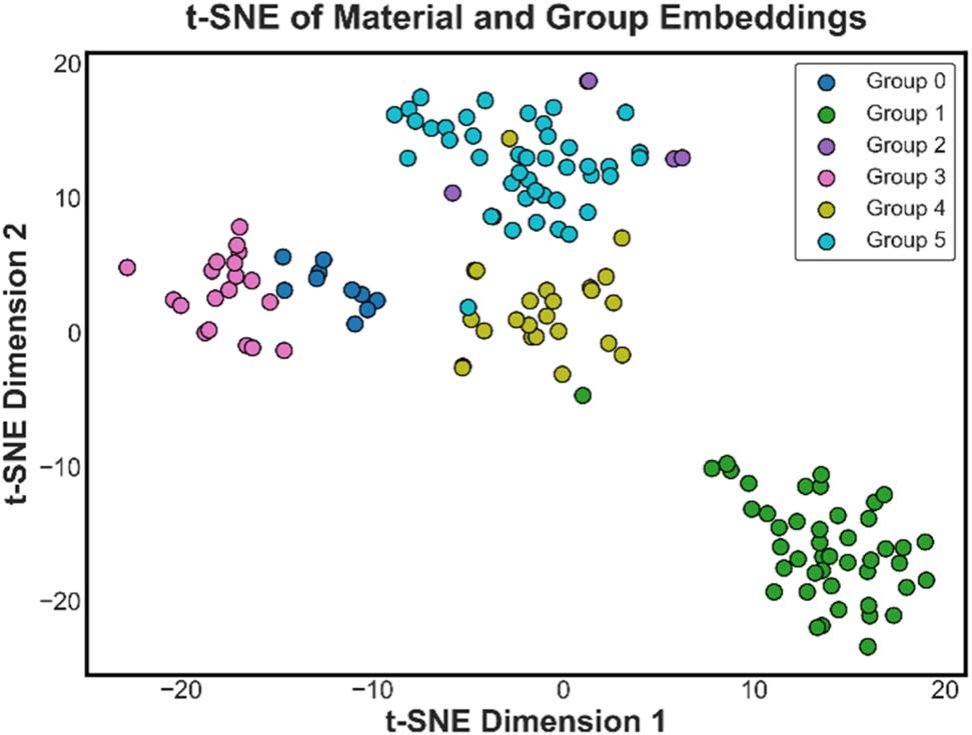
Embedding Analysis *via* t-distributed Stochastic Neighbour Embedding (t-SNE) of material and group embeddings.

It is crucial to understand that how the model internally organizes knowledge about materials, despite its predictive accuracy. To visualize, the learned material embeddings (*e*_*i*_ ∈ *R*^12^) were analyzed using a dimensionality reduction technique. Each embedding represents a compressed numerical fingerprint of a material's optical behaviour, learned directly through end-to-end training. To uncover latent structure, the 12-dimensional embedding matrix was given by the below eqn (11):

11 *E* = [*e*_1_, *e*_2_, …, *e*_*M*_] ∈ *R*^*M*×12^

was projected into a two-dimensional space using *t-distributed Stochastic Neighbour Embedding (t-SNE).* Formally, t-SNE seeks a mapping govern by below eqn (12):

12 *Z* = t-SNE(*E*) ∈ *R*^*M*×2^

where *Z* is the 2D projection of the material embeddings. Each point *z*_*i*_ ∈ *R*^2^ was then color-coded by its associated group label (group_id) and visualized. The t-SNE projection as shown in Fig. 3, reveals distinct groups for metals, semiconductors, and dielectrics, despite the model receiving no explicit chemical descriptors. This organization is physically intuitive, as optical constants are governed by a material's electronic structure, which is determined by its composition and lattice symmetry. Specifically, the observed patterns are consistent with known physical trends: the clustering of metals likely reflects shared high free-carrier densities, while semiconductors and dielectrics align according to their characteristic bandgap-driven transition profiles. These results suggest that the embedding space implicitly encodes functional similarities from the spectral response alone. We emphasize that these observations are qualitative interpretations intended to provide insight into the latent structure.

## 4. Results and discussion

Here, we conducted an ablation study comparing three strategies: (i) XGBoost, which represents a purely data-driven ensemble baseline, and also MLP without any physics regularization (ii) MLP with a fixed *β*_refl_, where the reflectance constraint is applied with a constant weight, and (iii) MLP with adaptive *β*_refl_, scheduling, where the constraint is introduced gradually during training.

### 4.1 Baseline comparison: MLP *vs.* XGBoost

**Fig. 4:**
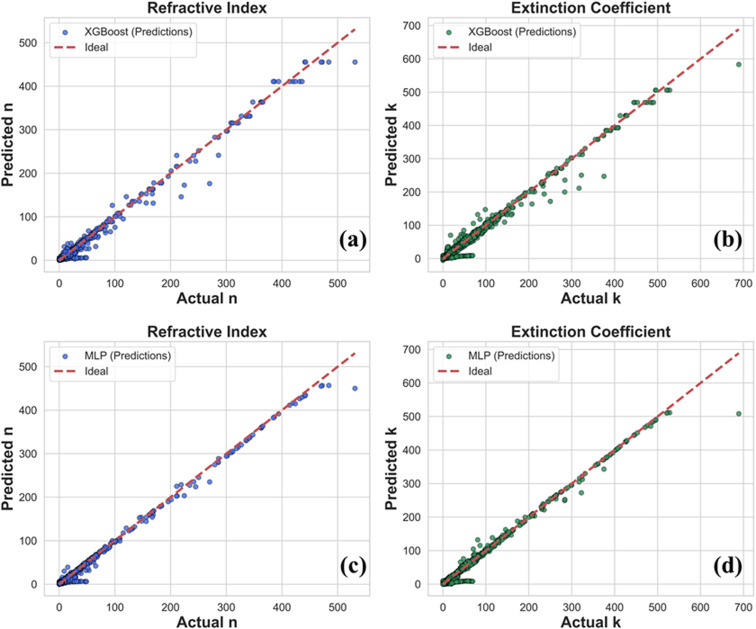
Comparison of predicted and actual values of (a) the refractive index (*n*(*λ*)) and (b) extinction coefficient (*k*(*λ*)) for XGBoost and (c) the refractive index (*n*(*λ*)) and (d) extinction coefficient (*k*(*λ*)) MLP. The dashed line shows the ideal case.

Firstly, to assess the model performance, we compared the predicted *versus* actual values of the refractive index (*n*(*λ*)) and extinction coefficient (*k*(*λ*)) using two approaches: the ensemble learning method XGBoost and an embedding-driven multilayer perceptron (MLP) based on deep learning. For the XGBoost, the material and group identifiers were provided as integer IDs and for MLP, material and group embeddings were used. Apart from this, we have also compared the embedding MLP model to the one-hot MLP (see SI, section S2), in addition to reduced accuracy, the one-hot representation increased the input dimensionality from 21 to 154 features, increasing the first-layer parameter count from 9526 (37.21 KB) to 16 226 (63.38 KB). Unlike one-hot encoding, which treats materials as independent categories, learned embeddings allow the model to capture similarity relationships in a continuous latent space, consistent with the structured clustering observed in Fig. 3. As illustrated in Fig. 4, both models generally follow the ideal trend (dashed red line), confirming that they capture the underlying relationship between wavelength, material, and optical constants. However, clear differences emerge in prediction accuracy. For the refractive index, shown in Fig. 4a and c, XGBoost displays noticeable deviations at higher values, indicating difficulty in fully capturing material-specific variations. In contrast, the MLP produces predictions that remain tightly clustered around the ideal line, reflecting its ability to better learn subtle nonlinear dependencies. A similar pattern is observed for the extinction coefficient in Fig. 4b and d. XGBoost predictions scatter more widely in the mid-to-high range, whereas the MLP aligns much more closely with the actual values, even in regions with sparser or more extreme data.

These observations are quantitatively supported by the error metrics summarized in Table 1. The MLP achieves lower RMSE and higher *R*^2^ scores for both *n*(*λ*) (RMSE = 0.84, *R*^2^ = 0.99) and *k*(*λ*)(RMSE = 1.50, *R*^2^ = 0.99), compared to XGBoost (*n*: RMSE = 1.46, *R*^2^ = 0.97; *k*: RMSE = 2.16, *R*^2^ = 0.97). Collectively, this demonstrates that the embedding-based MLP not only generalizes better but also provides more consistent predictions across a wide range of optical constants, highlighting the strength of spectral–material embeddings over traditional ensemble methods.

**Table 1:** Comparison of RMSE and *R*^2^ for two different models XGBoost and MLP

Model	Parameters	RMSE	*R* ^2^
XGBoost	*n*	1.4599	0.9714
	*k*	2.1625	0.9705
MLP	*n*	0.8436	0.9904
	*k*	1.5017	0.9857

### 4.2 Effect of reflectance-based regularization

**Fig. 5:**
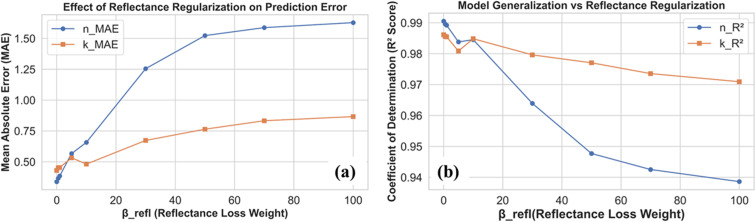
(a) Effect of Fresnel reflectance constraint (*β*_refl_, from 0−100) on the coefficient of determination (*R*^2^). (b) Effect of Fresnel reflectance constraint (*β*_refl_, from 0−100) on prediction error term (mean absolute error, MAE).

Next, we explored how adding a physics-based constraint influences the embedding-driven training process by introducing a reflectance-based loss term, controlled by the weight *β*_refl_. This term was designed to encourage the model to remain consistent with physical reflectance principles, but as shown in Fig. 5, it also reshapes the balance between prediction accuracy and generalization. As *β*_refl_. increases, the loss function shifts its priority toward physical regularization, promoting smoother, more stable spectral behavior that adheres to causal dispersion relations. In Fig. 5a, we see how the mean absolute error (MAE) for both the refractive index (*n*(*λ*)) and extinction coefficient (*k*(*λ*)) changes as *β*_refl_ increases. The errors gradually rise, with *n*(*λ*) being more strongly affected than *k*(*λ*). This suggests that while the reflectance constraint injects useful physical information into the training, it comes at the cost of slightly higher predictive error, particularly for refractive index values.

Fig. 5b shows the same trade-off from another perspective, using the coefficient of determination (*R*^2^). As *β*_refl_ grows, the *R*^2^ scores decline, most noticeably for *n*(*λ*). In other words, as the model focuses gives more importance to the reflectance constraint, it costs some of its capability to perfectly match the training data. As from the observation Table 2, these results highlight the subtle balance between data-driven accuracy and physics-based regularization. A moderate reflectance weight can help guide the model toward physically meaningful solutions, but overly strong regularization may hinder its predictive performance. Finding this balance is key to leveraging the strengths of both data and physics in a unified framework.

**Table 2:** Effect of Reflectance-Based Regularization on error metrics

*β*_refl_	MAE		RMSE		*R* ^2^	
	*n*	*k*	*n*	*k*	*n*	*k*
0.0	0.3206	0.4231	0.8436	1.5017	0.9904	0.9857
0.5	0.3198	0.4033	0.9222	1.4449	0.9857	0.9868
1.0	0.3488	0.4054	0.8641	1.5000	0.9899	0.9857
5.0	0.5117	0.4988	0.9854	1.5674	0.9869	0.9844
10.0	0.6110	0.4463	0.9804	1.4538	0.9871	0.9866
30.0	1.2206	0.6164	1.5682	1.6381	0.9670	0.9830
50.0	1.4819	0.7423	1.9352	1.8720	0.9498	0.9778
70.0	1.5756	0.8176	2.0600	2.0115	0.9431	0.9744
100.0	1.6448	0.8802	2.1785	2.1983	0.9364	0.9694

### 4.3 Adaptive scheduling of *β*_refl_

To overcome the trade-off between predictive accuracy and physical fidelity observed with fixed reflectance weighting, we developed an adaptive scheduling strategy for *β*_refl_. Instead of assigning a single fixed value, *β*_refl_ was allowed to evolve during training, gradually increasing from 0 up to a maximum weight of 30. Since Table 2 shows that accuracy begins to decline around *β*_refl_. = 30, this value was chosen as the optimal point for compare. Importantly, this ceiling can be tuned further depending on the desired balance between data-driven learning and physics regularization.

**Fig. 6:**
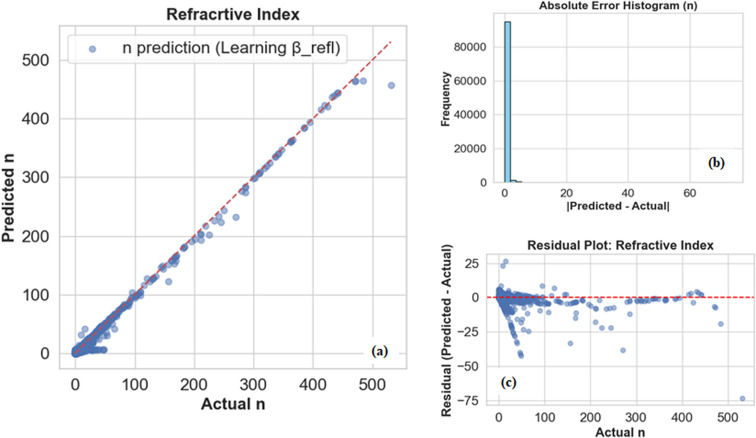
Refractive index analysis by deep learning model. (a) Refractive index prediction graph with adaptive *β*_refl_. (b) Absolute error histogram of refractive index. (c) Residual plot of refractive index.

As shown in Fig. 6a–c, this adaptive scheduling produced significant improvements for refractive index (*n*(*λ*)) predictions. The scatter plot confirms that the predicted values align almost perfectly with the ideal diagonal, while the residuals remain tightly centered around zero without exhibiting systematic drift. The error histogram further highlights that most deviations are minimal, demonstrating that the model preserved the predictive sharpness of the unconstrained baseline while still benefiting from the gradual incorporation of the reflectance constraint.

**Fig. 7:**
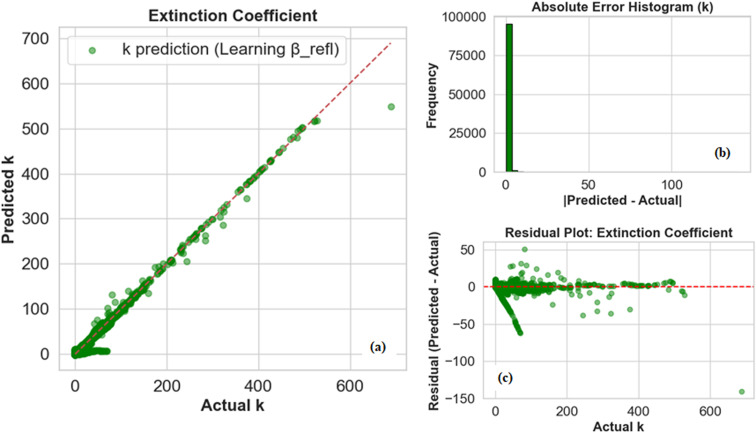
Extinction coefficient analysis by deep learning model. (a) Extinction coefficient prediction graph with adaptive *β*_refl_. (b) Absolute error histogram of extinction coefficient. (c) Residual plot of extinction coefficient.

For the extinction coefficient (*k*(*λ*)), the improvements are equally clear in Fig. 7a–c. Predictions remain closely aligned with the actual values across the full test range, including high-absorption regions where fixed *β*_refl_ previously led to systematic underestimation. The residual plot shows a well-balanced distribution around zero, and the histogram indicates that large errors are rare. This outcome underscores the strength of the adaptive strategy: by introducing the reflectance constraint only after the model has already captured the core data-driven mapping, we prevent the physics term from overwhelming the learning dynamics too early. Overall, the adaptive *β*_refl_ strategy achieves the optimal compromise: it preserves high predictive accuracy for both *n* and *k*, while simultaneously improving physical consistency with reflectance constraints. By capping *β*_refl_ at 30, the model maintains a stable trade-off between accuracy and physics, though this upper limit can be flexibly adjusted if stronger or weaker physical enforcement is required for specific applications. This staged integration of physics demonstrates the power of embedding domain knowledge into deep learning models in a gradual, controlled manner, rather than enforcing it rigidly from the outset.

**Fig. 8:**
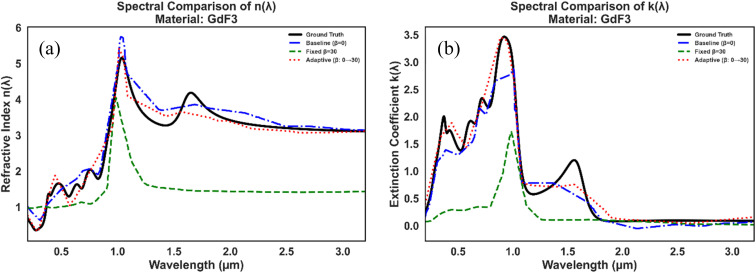
Spectral comparison of predicted and ground-truth optical constants for GdF_3_: (a) refractive index *n*(*λ*)and (b) extinction coefficient *k*(*λ*). Results are shown for the baseline model (*β* = 0), fixed reflectance constraint (*β* = 30), and adaptive reflectance constraint (β: 0 → 30).

From the Table 3, we observed that MLP model with adaptive *β*_refl_ performs better as compared to the MLP model with fixed *β*_refl_. To compare the two models, we set *β*_refl_ to 30, as Table 2 shows that predictive accuracy starts to decline around this value. So, for the fixed *β*_refl_ value, the model already exhibits strong predictive capabilities, with MAE values of 1.22 for the refractive index (*n*(*λ*))and 0.62 for the extinction coefficient (*k*(*λ*)), and RMSE values of 1.57 and 1.64, respectively. The *R*^2^ scores of 0.967 *n*(*λ*) and 0.983 *k*(*λ*) indicate that the model captures most of the variance in the data. However, implementing an adaptive *β*_refl_ (0 to 30) schedule leads to marked improvements, particularly for the refractive index. The MAE for *n*(*λ*) drops dramatically from 1.22 to 0.34, a 72% reduction, while RMSE decreases by 45%, reaching 0.86. Correspondingly, *R*^2^ improves to 0.990, signalling near-ideal predictive performance. The extinction coefficient *k*(*λ*) also benefits from the adaptive approach, with MAE decreasing by 32% (0.62 to 0.42) and RMSE by 11% (1.64 to 1.46), alongside a modest *R*^2^ increase to 0.986. Additionally, Fig. 8 presents spectral comparisons of *n*(*λ*) and *k*(*λ*) for GdF_3_, showing that the adaptive incorporation of the reflectance constraint better preserves spectral features and improves agreement with ground truth trends, while the fixed constraint introduces noticeable bias. This underscores the benefit of gradually embedding physical knowledge into data-driven models rather than enforcing it rigidly.

**Table 3:** Comparison between *β*_refl_. weightage for fixed value (30) and adaptive value (0 to 30)

*β*_refl_	MAE		RMSE		*R* ^2^	
	*n*	*k*	*n*	*k*	*n*	*k*
Fixed 30	1.2206	0.6164	1.5682	1.6381	0.9670	0.9830
Adaptive 30	0.3423	0.4179	0.8638	1.4587	0.9900	0.9865

To further contextualize the performance of our optimized model, we benchmarked it against a recent regression-based study by Einabadi and Mashkoori (2024),^24^ who investigated refractive index prediction of inorganic compounds using machine learning employed SVR, GPR, and Random Forest Regression models using 21 handcrafted inorganic descriptors on a dataset of 272 samples, reporting a best-case MAE of 0.156. However, our base model achieves an MAE (*n*) of 0.3206 and MAE (*k*) of 0.4231 across a substantially larger and more diverse material library. While the absolute MAE is higher, the two approaches differ fundamentally: the prior work relies on domain-specific handcrafted features, whereas our framework is descriptor-free and domain-agnostic, learning material representations directly through embeddings.

To evaluate the impact of dataset imbalance as shown in Fig. 1, we performed a stratified analysis across six primary material classes, with detailed metrics provided in supplementary information (Table S3). Despite substantial differences in sample counts, the model maintains consistent predictive performance across both majority and minority groups. Notably, the Metal class, representing only ∼8% of the test set, achieves the highest accuracy (*R*^2^(*n*) = 0.9970, *R*^2^(*k*) = 0.9931), confirming that performance is not biased toward high-density categories. However, structurally diverse groups such as Oxides exhibit comparatively higher RMSE, overall trends remain physically consistent. In the case of Oxides, *R*^2^(*n*) = 0.4773 and *R*^2^(*k*) = 0.2259 which shows that performance of model drops significantly. This behavior reflects the inherent complexity of oxides rather than dataset imbalance alone. Unlike other material classes, oxides exhibit substantial structural and electronic diversity, and the model notably struggles with their vast polymorphism, where the same composition can exist in multiple phases with very different optical responses. Since the model relies only on wavelength and material identifiers, the embeddings are forced to represent a wide range of underlying factors such as phase, defects, and composition, making the learning task particularly challenging for this class. Along with limited embedding capacity and the absence of global physical constraints, together restrict the ability of model to accurately capture oxide-specific optical behavior. Apart from this case, the results demonstrate that the embedding-driven architecture mitigates long-tailed distribution effects and ensures balanced generalization across material families.

## 5. Conclusion

In this study, we introduced an embedding-driven deep learning framework for predicting wavelength-dependent optical constants using only material identity and wavelength as inputs. When benchmarked against XGBoost, the multilayer perceptron (MLP) architecture demonstrated a stronger ability to capture material-specific nonlinearities, leading to higher accuracy for both the refractive index *n*(*λ*) and extinction coefficient *k*(*λ*). Incorporating a reflectance-based constraint further emphasized the value of embedding physical knowledge into data-driven models. While applying a fixed *β*_refl_. improved physical interpretability, particularly in high-absorption regimes, it also came with a slight reduction in predictive precision. Since Table 2 shows that accuracy begins to decline around *β*_refl_. = 30, this value was chosen as the optimal point for comparison. To address the accuracy-interpretability trade-off, we implemented an adaptive *β*_refl_ scheduling strategy. This yielded a substantial performance gain, reducing the MAE for *n*(*λ*) by 72% and RMSE by 45%, while also improving predictions for *k*(*λ*) with 32% and 11% reductions in MAE and RMSE, respectively. By gradually enforcing Fresnel-consistent reflectance, this adaptive strategy preserved the strong predictive power of the unconstrained model while aligning it more closely with physical principles.

Overall, the proposed framework demonstrates good predictive capability across most material classes and spectral ranges. However, reduced accuracy is observed for oxide materials, reflecting their high compositional and spectral diversity, which is not fully captured by the current embedding-based representation. Addressing this limitation through improved feature representations or class-specific modelling remains an important direction for future work. Despite this, the embedding-based approach provides a compact and transferable latent space that captures meaningful relationships between materials. The integration of adaptive physics-informed regularization further illustrates how machine learning models can balance predictive accuracy with physical consistency, offering a promising tool for optical modelling, materials screening, and data-driven design.

## Conflicts of interest

There are no conflicts to declare.

## Supplementary Material

RA-016-D6RA00706F-s001

## Data Availability

Instructions, example scripts, and used data set used in finetuning can be found at https://github.com/Sakshich1912/RINFO. Data set for the neural network were curated from https://refractiveindex.info/. Supplementary information (SI): sensitivity analysis of embedding dimensionality, comparison with One-Hot encoding and performance analysis across material classes. See DOI: https://doi.org/10.1039/d6ra00706f.

## References

[cit1] Fox M. , Optical Properties of Solids, Oxford Master Series in Condensed Matter Physics, Oxford Univ. Press, Oxford, 2nd edn, 2012

[cit2] Palik E. D. , Handbook of Optical Constants of Solids, Academic Press, 1998, Vol. 3

[cit3] Yu P. Y. , and Cardona M., Fundamentals of Semiconductors: Physics and Materials Properties, Graduate Texts in Physics, Springer, Berlin, Heidelberg, 2010. 10.1007/978-3-642-00710-1

[cit4] Atwater H. A., Polman A. (2010). Plasmonics for Improved Photovoltaic Devices. Nat. Mater..

[cit5] Born M. , and Wolf E., Principles of Optics: Electromagnetic Theory of Propagation, Interference and Diffraction of Light, Cambridge University Press, Cambridge, 1999, 7th edn. doi: 10.1017/CBO9781139644181

[cit6] Johnson P. B., Christy R. W. (1972). Optical Constants of the Noble Metals. Phys. Rev. B.

[cit7] Azzam R. M. A. , Ellipsometry and Polarized Light, North-Holland Pub. Co., Amsterdam; New York, New York : sole distributors for the U.S.A. and Canada, Elsevier North-Holland, 1977

[cit8] Tompkins H. , and Irene E. A., Handbook of Ellipsometry, William Andrew, 2005

[cit9] Curtarolo S., Setyawan W., Hart G. L. W., Jahnatek M., Chepulskii R. V., Taylor R. H., Wang S., Xue J., Yang K., Levy O., Mehl M. J., Stokes H. T., Demchenko D. O., Morgan D. (2012). AFLOW: An Automatic Framework for High-Throughput Materials Discovery. Comput. Mater. Sci..

[cit10] Butler K. T., Davies D. W., Cartwright H., Isayev O., Walsh A. (2018). Machine Learning for Molecular and Materials Science. Nature.

[cit11] Raccuglia P., Elbert K. C., Adler P. D. F., Falk C., Wenny M. B., Mollo A., Zeller M., Friedler S. A., Schrier J., Norquist A. J. (2016). Machine-Learning-Assisted Materials Discovery Using Failed Experiments. Nature.

[cit12] Schmidt J., Marques M. R. G., Botti S., Marques M. A. L. (2019). Recent Advances and Applications of Machine Learning in Solid-State Materials Science. npj Comput. Mater..

[cit13] Jacobs R., Schultz L. E., Scourtas A., Schmidt K., Price-Skelly O., Engler W., Foster I., Blaiszik B., Voyles P. M., Morgan D. (2024). Machine Learning Materials Properties with Accurate Predictions, Uncertainty Estimates, Domain Guidance, and Persistent Online Accessibility. Mach. Learn. Sci. Technol..

[cit14] Ramprasad R., Batra R., Pilania G., Mannodi-Kanakkithodi A., Kim C. (2017). Machine Learning in Materials Informatics: Recent Applications and Prospects. npj Comput. Mater..

[cit15] Rajendra P., Girisha A., Gunavardhana Naidu T. (2022). Advancement of Machine Learning in Materials Science. Mater. Today Proc..

[cit16] Pilania G., Wang C., Jiang X., Rajasekaran S., Ramprasad R. (2013). Accelerating Materials Property Predictions Using Machine Learning. Sci. Rep..

[cit17] Ward L., Agrawal A., Choudhary A., Wolverton C. (2016). A General-Purpose Machine Learning Framework for Predicting Properties of Inorganic Materials. npj Comput. Mater..

[cit18] Isayev O., Fourches D., Muratov E. N., Oses C., Rasch K., Tropsha A., Curtarolo S. (2015). Materials Cartography: Representing and Mining Materials Space Using Structural and Electronic Fingerprints. Chem. Mater..

[cit19] Mikolov T. , Chen K., Corrado G. and Dean J., Efficient Estimation of Word Representations in Vector Space, International Conference on Learning Representations, 2013

[cit20] Mol2vec: Unsupervised Machine Learning Approach with Chemical Intuition | Journal of Chemical Information and Modeling, https://pubs.acs.org/doi/10.1021/acs.jcim.7b00616, (accessed 2025-10-22) 10.1021/acs.jcim.7b00616 29268609

[cit21] Xie T. (2018). Crystal Graph Convolutional Neural Networks for an Accurate and Interpretable Prediction of Material Properties. Phys. Rev. Lett..

[cit22] Maaten L. v. d., Hinton G. (2008). Visualizing Data Using T-SNE. J. Mach. Learn. Res..

[cit23] Polyanskiy M. N. (2024). Refractiveindex.Info Database of Optical Constants. Sci. Data.

[cit24] Einabadi E., Mashkoori M. (2024). Predicting Refractive Index of Inorganic Compounds Using Machine Learning. Sci. Rep..

