# Supplementary material for: Embedding-driven physics informed neural network for predicting optical constants across materials
Source: RSC Adv. 2026 May 14;16(28):25747–57. doi: 10.1039/d6ra00706f (PMC13182532; doi:10.1039/d6ra00706f)
Supplement: RA-016-D6RA00706F-s001 [file RA-016-D6RA00706F-s001.pdf]

Supplementary Information (SI) for RSC Advances.
This journal is © The Royal Society of Chemistry 2026

Supplementary Information

Embedding-Driven Physics Informed Neural Network for Predicting Optical Constants Across Materials

Sakshi Choudhary[1,#], Ravi Kumar[1], Annapureddy Venkateswarlu[2], Salla Gangi Reddy[1,*]
[1]Department of Physics, SRM University-AP, Amaravati 522 240, Andhra Pradesh, India
[2] *Department of Physics, National Institute of Technology, Tiruchirappalli, Tamil Nadu, India.*
[#] sakshi.a@srmap.edu.in, [*]gangireddy.s@srmap.edu.in

S1. Sensitivity Analysis of Embedding Dimensionality:

To determine the optimal embedding dimensionality, a systematic ablation study was conducted. The material embedding dimension was varied across {4, 8, 12, 16, 32}, and the group embedding dimension across {2, 4, 8, 16}, while keeping all other architectural parameters and training conditions fixed. All the results were summarized in the Table S1.

Based on the ablation results in Table S1, the embedding dimensions were selected by balancing predictive accuracy, generalization, and parameter efficiency. Very small embeddings (e.g., material embedding = 4 or group embedding = 2) already achieve high R^2, but consistently show higher test RMSE, indicating limited capacity to capture subtle wavelength-dependent dispersion variations. Increasing the dimensions improves error metrics, confirming the need for richer material and group representations. The configuration (material embedding = 12, group embedding = 8) provides the most balanced performance, achieving low test errors (RMSE (n) test = 0.8412, RMSE (k) test = 1.4260) with strong generalization (R^2 (n) test ≈ 0.9905, R^2 (k) test ≈ 0.9872) and a minimal training and testing gap. While larger embeddings (e.g., 16, 16 or 32, 16) offer only marginal gains (<1–2%), they increase parameter count and show mild overfitting. Overall, (12,8) offer an optimal trade-off between expressiveness and complexity, therefore selected for the final model.

Table S1. Effect of embedding dimensionality on test performance.

**Material Embedding**	**Group Embedding**	**RMSE (*n*)**		**R^2 (*n*)**		**RMSE (*k*)**		**R^2 (*k*)**	
		Training	Testing	Training	Testing	Training	Testing	Training	Testing
4	2	0.8109	0.8510	0.9907	0.9903	1.3943	1.4715	0.9877	0.9863
	4	0.8082	0.8259	0.9907	0.9909	1.3841	1.3983	0.9879	0.9877
	8	0.8370	0.8885	0.9901	0.9894	1.3951	1.4320	0.9877	0.9870
	16	0.7881	0.8441	0.9912	0.9905	1.3798	1.3934	0.9879	0.9877
8	2	0.8189	0.8571	0.9905	0.9902	1.3932	1.4392	0.9877	0.9869
	4	0.9631	0.9832	0.9868	0.9871	1.5128	1.5538	0.9855	0.9848
	8	0.8250	0.8661	0.9903	0.9900	1.4196	1.4770	0.9872	0.9862
	16	0.7806	0.8395	0.9914	0.9906	1.3748	1.4519	0.9880	0.9867
**12**	2	0.9328	0.9530	0.9877	0.9878	1.4721	1.5300	0.9863	0.9852
	4	1.0785	1.1286	0.9835	0.9829	1.6644	1.7619	0.9825	0.9804
	**8**	**0.8300**	**0.8412**	**0.9902**	**0.9905**	**1.4185**	**1.4260**	**0.9873**	**0.9872**
	16	0.8057	0.8459	0.9908	0.9904	1.3956	1.5138	0.9877	0.9855
16	2	0.8000	0.8922	0.9909	0.9893	1.3882	1.4532	0.9878	0.9867
	4	1.0355	1.0644	0.9848	0.9848	1.6796	1.7625	0.9821	0.9804
	8	0.8912	0.9120	0.9887	0.9889	1.4657	1.5323	0.9864	0.9852
	16	0.8526	0.9560	0.9897	0.9878	1.3898	1.4999	0.9878	0.9858
32	2	0.8737	0.9474	0.9892	0.9880	1.4390	1.5919	0.9869	0.9840
	4	0.8798	0.9040	0.9890	0.9891	1.4635	1.5330	0.9864	0.9852
	8	0.7874	0.8302	0.9912	0.9908	1.3855	1.4569	0.9878	0.9866
	16	0.8122	0.9424	0.9906	0.9881	1.4044	1.4753	0.9875	0.9863

Bold values correspond to the selected configuration.

S2. Comparison with One-Hot Encoding:

To evaluate the advantage of embeddings over one-hot, a baseline MLP was implemented using one-hot encoded material (147-dimensional) and group (6-dimensional) vectors, increasing the total input dimension from 21 (embedding-based model) to 154. This expansion nearly doubled the number of parameters in the first dense layer (16,226 vs. 9,526; 63.38 KB vs. 37.21 KB). One-hot model produced noticeably higher test errors (RMSE *(n)* = 1.3144, RMSE *(k)* = 1.9147) compared to the embedding-based model (RMSE *(n)* = 0.8436, RMSE *(k)* = 1.5017). This indicates poorer generalization despite increased model size. The performance gap arises because one-hot encoding treats each material as completely independent, preventing the model from learning shared structure. In contrast, learned embeddings map materials into a continuous latent space, allowing related materials to share dispersion characteristics. This improves parameter efficiency, reduces overfitting risk, and enhances predictive accuracy.

Table S2. Comparison between one-hot encoding and learned embeddings.

	**Input Dimension**	**First layer parameters**	**RMSE *(n)***	**RMSE *(k)***
**One-hot encoding**	154	16,226 (size = 63.38 KB)	1.3144	1.9147
**Learned embeddings**	21	9,526 (size = 37.21 KB)	0.8436	1.5017

S3: Performance Analysis Across Material Classes

To further examine the impact of dataset imbalance, we performed a detailed stratified evaluation across six primary material groups: semiconductors, oxides, metals, dielectrics, nitrides, and others. The corresponding per-group RMSE and R^2 metrics for both $n$and $k$predictions are summarized in Supplementary Table S3. Despite substantial variation in test sample counts (ranging from 2,204 for nitrides to 37,054 for semiconductors), the model demonstrates stable and physically consistent generalization across all categories. Notably, the Metal group, representing approximately 8% of the test set, achieves the highest predictive accuracy (n - $R^2 = 0.9970$, k - $R^2 = 0.9931$), indicating that performance is not dominated by majority classes. Dielectrics and semiconductors also exhibit strong predictive fidelity, while nitrides maintain robust absorption prediction despite limited data density. The comparatively higher RMSE observed for oxides is consistent with their broader compositional, structural, and bonding diversity, rather than solely reflecting data imbalance. These results confirm that the embedding-driven architecture preserves predictive stability and physical consistency even under pronounced class imbalance.

Table S3. Performance Analysis Across Material Groups:

**Material Group**	**Test Samples**	**RMSE *(n)***	**RMSE *(k)***	**R^2 *(n)***	**R^2 *(k)***
Semiconductor	37,054	0.4735	0.4551	0.8066	0.8313
Oxide	25,083	1.1510	2.1106	0.4773	0.2259
Other	17,659	0.4781	0.5964	0.8370	0.7745
Metal	8,175	1.5753	3.2774	0.9970	0.9931
Dielectric	6,888	0.2038	0.3416	0.9607	0.8735
Nitride	2,204	1.1542	1.4454	0.7753	0.8973
